# Serum lactate and LDH are related with theta and gamma activities in bipolar disorder: a band-specific metabolic coupling

**DOI:** 10.3389/fpsyt.2025.1695916

**Published:** 2025-10-09

**Authors:** Sermin Kesebir, Rüştü Murat Demirer

**Affiliations:** ^1^ Üsküdar University School of Medicine Department of Psychiatry Üsküdar University, NPİstanbul Brain Hospital, İstanbul, Türkiye; ^2^ Natural and Engineering Sciences, Üsküdar University, İstanbul, Türkiye

**Keywords:** bipolar disorder, mitochondrial dysfunction, lactate, LDH, metabolic syndrome, EEG

## Abstract

**Objective:**

Bipolar disorder includes features of a biphasic energy disregulation. Lactate and LDH have been suggested as biomarkers for mitochondrial dysfunction, which has a role in its etiology. This study aims to examine the correlation between electrophysiological brain dynamics, quantified by Entropy Doubling and Ruzsa Distance derived from EEG data, and peripheral lactate and lactate dehydrogenase (LDH) levels in patients with bipolar disorder in remission.

**Methods:**

In this study, 20 individuals diagnosed with Bipolar Disorder Type I following DSM-V criteria were consecutively assessed throughout their remission phase while attending our outpatient unit for routine evaluations. Metabolic syndrome and the usage of conventional antipsychotics serves as an exclusion criterion. We examined serum LDH and lactate levels and did EEGs. All EEG data is arranged with a sample rate of 125 Hz. The additive combinatorial entropy of the electrodes is what makes up the entropic Rusza Distance. The Hilbert-based Entropy Doubling approach was used to process the analytical signals for each EEG channel.

**Results:**

Energy dysregulation includes theta and gamma frequency bands, both in relation to lactate and LDH. Lactate and LDH levels in the F7 theta band were linearly correlated. A negative correlation was found between the levels of lactate and LDH levels in the O1, Fz, and Cz gamma bands.

**Conclusion:**

Our findings suggest that there is a unique relationship between electrophysiological brain dynamics and mitochondrial dysfunction mediated metabolic stress in bipolar disorder.

## Introduction

The brain’s high-energy activity necessitates glucose, a 6-carbon monosaccharide, for ATP generation (1). Glucose penetrates the parenchyma via glucose transporter 1 (GLUT1) located in the endothelial cells of the blood-brain barrier. Neurons uptake it through GLUT3 and GLUT4. Monocarboxylate transporters (MCTs), located in brain endothelial cells, neurons, and glia, facilitate the utilization of alternate energy substrates, including lactate.

Lactate is a three-carbon monosaccharide that can be produced and released by a variety of cells, including immune cells. The discovery of the lactate shuttle revealed that lactate is not a waste product but a metabolic fuel (2, 3). It is also an intercellular messenger and plays a role in gene expression (4). In mitochondria, the metabolism of two molecules of lactate yields 30 ATP, while one molecule of glucose yields another 2 ATP through glycolysis. In neurons, the majority of ATP is consumed to drive ion pumps for signaling and conduction.

Lactate for ATP generation is associated with oxidative phosphorylation in the cell’s mitochondria (4). This metabolic process necessitates the conversion of lactate to pyruvate by lactate dehydrogenase (LDH), a bidirectional redox enzyme. The conversion is facilitated by the tricarboxylic acid cycle (TCA) and molecular oxygen, which acts as the terminal electron acceptor in the respiratory chain. Consequently, cellular oxygen consumption rises linearly with the complete oxidation of lactate.

Lactate is conveyed from the circulation, astrocytes, oligodendrocytes, and activated microglia to neurons (1). This amount is significantly elevated relative to the output of neuronal glucose metabolism. At this juncture, it is crucial to ascertain whether the transferred molecule serves as a more fundamental source of pyruvate.

A key goal is to elucidate the relationship between lactate oxidation and cortical functions such as perception, motor activity, and memory formation, and its role in fueling neuronal excitation and signal transmission. This exciting topic has been studied ex vivo in hippocampal slice preparations using electrical stimulation, optogenetic tools, and receptor-ligand applications. Electrophysiological experiments allowing the induction of different neural networks have shown that, in the absence of glucose, only lactate disrupts gamma and theta-gamma oscillations, which require high energy requirements (5–7), during which time the cerebral oxygen metabolic rate is fully regulated. This disruption is characterized by moderate hyperexcitability and reflects excitation-inhibition dysregulation. This dysregulation is suppressed by increasing the glucose fraction of the energy substrate. In contrast, lactate alone preserves the lower-energy, intermittent sharp wave activity when the cerebral oxygen metabolic rate is around 65%. Another consideration is that lactate slows neurotransmission in pyramidal cells and fast-firing GABAergic interneurons by reducing neurotransmitter release from presynaptic terminals, whereas in the axon, the generation and propagation of action potentials are regular.

The aim of this study is to investigate the relationship between electrophysiological brain dynamics obtained from EEG signals and peripheral lactate and lactate dehydrogenase (LDH) levels measured by Entropic Ruzsa Distance and Entropy Doubling methods in patients with bipolar disorder in remission.

Indeed, bipolar disorder exhibits the characteristics of a biphasic energy disorder (8–10). It is characterized by depressive and manic episodes, characterized by mood swings, and increased and decreased psychomotor activity. Furthermore, cognitive impairment is also observed during periods of remission in these patients (11). Mitochondrial dysfunction is a current and important area of ​​research in the etiology of this neuroprogressive disorder (12, 13).

The initial study conducted in 1990 by Swartz and Breen revealed that serum LDH levels during manic episodes were elevated compared to healthy controls (14). It has been demonstrated that bipolar cases can be differentiated from unipolar cases among 2,470 inpatients in Shanghai (15). A comparable outcome was observed in a concurrent study involving 261 adolescent cases (16).

In bipolar cases, elevated lactate levels were observed in the brain via Magnetic Resonance Spectroscopy (MRS) in six studies and in cerebrospinal fluid (CSF) in two studies (17). There are two positive and two negative results in peripheral measurements. Guo and colleagues indicated that increased serum lactate correlates with depressive episodes (18). Vieira et al. demonstrated the reversibility of this elevation with lithium (19). Serum lactate levels, indicative of bipolar disorder, were elevated in patients relative to healthy controls, whereas cct-mtDNA levels, another proposed biomarker for mitochondrial dysfunction, showed no significant difference (20). It has been observed to correlate with lactate in bipolar cases, but not in healthy controls, and to exhibit a negative correlation with depressive symptoms.

Animal studies indicating elevated lactate levels in the brain propose that, alongside bipolar disorder, it may function as a transdiagnostic endophenotype associated with cognitive deficits in schizophrenia, autism, epilepsy, and Alzheimer’s (21). The shared characteristic of the five studies involving 2,294 animals is the correlation between elevated lactate levels and diminished working memory performance.

## Methods

In this study, 20 patients diagnosed with Bipolar Disorder Type I according to DSM-V were consecutively evaluated during their routine outpatient follow-up visits to our outpatient unit. Metabolic syndrome was an exclusion criterion because multivariate regression analyses have shown that lactate is associated with triglycerides, blood glucose, and systolic and diastolic blood pressure (22). Mitochondrial markers differ between patients with metabolic syndrome with normal and elevated lactate levels. Another exclusion criterion was the use of typical antipsychotics, as a potential elevation in creatine kinase would affect lactate and LDH levels (14).

Our university’s ethics committee gave us the green light, and we used the project-based research grant from our university as the source. We examined serum LDH and lactate levels and did EEGs on patients who gave their informed consent.

The additive combinatorial entropy of the electrodes is what makes up the entropic Rusza Distance (23). The Hilbert-based Entropy Doubling approach was used to process the analytical signals for each EEG channel.

All EEG data was recorded in a quiet, subtly lit room, in sitting position, with eyes closed. Nineteen scalp electrodes were placed according to the 10–20 system. Linked mastoid electrodes (A1–A2) were used for reference. EEG was recorded at a sample rate of 125 samples/s. Recording time was 3 min. Impedances for each electrode referring channels were kept below 30 kΩ. EEG processed offline for artifact rejection. A high pass filter was applied at 0.1 Hz and a low pass filter was applied at 70 Hz.

### Mathematical approach

We employed entropy-based metrics recently formalized in additive combinatorics to quantify hidden structural redundancy in EEG signals. We go through the steps, histogram definition, convolution, entropy-doubling equations and zero-phase guarantee.

#### EEG preprocessing and band definition

Raw EEG recordings were imported from EDF format and preprocessed using the FieldTrip toolbox (24). Data were sampled at 125 Hz, which was the acquisition rate for all patients. Signals were re-referenced to the common average reference after removal of non-EEG channels.

Continuous data were segmented into non-overlapping epochs of 2s (250 samples per epoch). Each epoch was then bandpass filtered into canonical frequency bands. Filters were designed as linear-phase finite impulse response (FIR) filters using the windowed sinc method, implemented with zero-phase forward-backward application (filtfilt command in MATLAB/FieldTrip) to avoid phase distortion (25). Stopband attenuation was at least 40 dB, with transition widths of ∼10% of the passband edge frequency.

#### Entropy doubling

Entropy doubling features were computed by adapting the entropic doubling constant


$$\sigma_{\text{ent}}[X]=\text{exp}(H(X_{1}+X_{2})-H(X))$$

where $X_{1},X_{2}$ are independent copies of the discretized EEG signal and H(·) denotes Shannon entropy, following the formulation (26, 27). For each EEG epoch, the empirical distribution $p_{X}$ (histogram) is estimated from amplitude or phase samples, and entropy is computed for both the original and doubled distributions. This procedure highlights repetitions and hidden organizational patterns beyond random variability.

#### Entropic Ruzsa distance

We further evaluated the entropic Ruzsa distance based on the same classical Shannon entropy.


$$d_{\text{ent}}(X,Y)=H(X^{\prime }-Y^{\prime })-\frac{1}{2}H(X^{\prime })-\frac{1}{2}H(Y^{\prime })$$

where $X^{\prime },Y^{\prime }$ are independent copies of X,Y , respectively. In our adaptation, EEG epochs were discretized into empirical probability distributions, and pairwise entropic distances were computed between channels in a 10–20 electrode system. This measure captures the degree of independence or redundancy among brain regions.

#### Theoretical link between entropy doubling and entropic Ruzsa distance

The two measures are mathematically related by


$$\sigma_{\text{ent}}[X]=\text{exp}(d_{\text{ent}}(X,-X))$$

which provides a unified framework for assessing both within-channel structural complexity and cross-channel dependence in EEG dynamics.

#### Smoothing window

We stabilize empirical distributions within each epoch $E_{k}=[t_{k},t_{k}+T)$ , apply a symmetric kernel $g_{\tau }(t)$ :


$${\tilde{a}}_{b}(t)=(g_{\tau }*a_{b})(t)$$

$${\tilde{\phi }}_{b}(t)=\text{unwrap}((g_{\tau }*\phi_{b})(t))$$

#### Vectorization operator

For each epoch $E_{k}$ with sample grid ${\left\{t_{j}\right\}}_{j=1}^{N}$


$$\begin{array}{l} a_{b,k}={\left\{{\tilde{a}}_{b}(t_{j})\right\}}_{j=1}^{N}\in {\mathbb{R}}^{N}\\ \phi_{b,k}={\left\{{\tilde{\phi }}_{b}(t_{j})\right\}}_{j=1}^{N}\in {\mathbb{R}}^{N} \end{array}$$

- where ${\left\{t_{j}\right\}}_{j=1}^{N}$ are the discrete sampling points inside epoch $E_{k}$ $a_{b,k}$ is simply the vector of amplitude samples in epoch $E_{k}$ .
-
$\phi_{b,k}$ is the vector of phase samples in epoch $E_{k}$ .

Independent-copy property is guaranteed on only circular shifts. We generate two approximately independent surrogates by applying circular shifts to the amplitude and phase vectors within each epoch.

- Epoch integer index k : each trial or segment is labeled by k .
- Sample index n∈{0,…,N−1} : denotes the discrete time sample inside epoch $E_{k}$ . For example 2 sec. epoch corresponds to N=250 samples in the case of sampling frequency of $f_{s}=125\;Hz.$ Shift amount s is an integer (uniformly chosen in [1,N−1] ) representing how many samples we circularly rotate.

Formally, for the amplitude vector $a_{b,k}[n]$ :


$$\left(T_{s}a_{b,k})[n]=a_{b,k}[(n-s)\text{mod}N\right]$$

and for the phase vector $\phi_{b,k}[n]$ :


$$\left(T_{s}\phi_{b,k})[n]=\phi_{b,k}[(n-s)\text{mod}N\right]$$

we define surrogates:


$$X_{1}^{\left(a\right)}=T_{s_{1}}a_{b,k}$$

$$X_{2}^{\left(a\right)}=T_{s_{2}}a_{b,k}$$

We can write the surrogates similar analogously for phase. This avoids block-bootstrap operators (28). The circular shift ensures all samples are preserved, only permuted. Using two different random shifts creates two copies of the same distribution that are decorrelated enough to act as independent samples. This procedure avoids block-bootstrap resampling (no cutting/rejoining of time series) while preserving amplitude and phase statistics within each 2-sec epoch.

#### Distribution-level convolution

Let X denote a vector of values from one epoch, either amplitude samples ${\left\{a_{b,k}[n]\right\}}_{n=1}^{N}$ or phase samples ${\left\{\varphi_{b,k}[n]\right\}}_{n=1}^{N}$ . To estimate its probability distribution, we construct an empirical histogram with $n_{\text{Bins }}=50$ equal-width bins (linear for amplitude, circular for phase). The normalized histogram yields the discrete distribution


$$p_{X}[j]=\frac{1}{N}\#\{n:X[n]\in {\text{bin}}_{j}\},\;j=1,\ldots ,n_{\text{Bins }}$$

This $p_{X}$ represents the probability distribution of a single surrogate copy of the data. Since $X_{1}$ and $X_{2}$ are generated by independent circular shifts of the same signal, their distributions are identical, both equal to $p_{X}$ . The distribution of their sum is then defined by discrete convolution: $p_{S}=p_{X}*p_{X}$


$$p_{S}[k]=\sum_{i}p_{X}[i]p_{X}[k-i]$$

Entropy is then estimated for both $p_{X}$ and $p_{S}$ using the Shannon estimator, yielding the entropy-doubling statistic. This step follows the entropy-doubling framework (27).

#### Entropy-doubling estimators

With equal-width bins ( n Bins =50 for amplitude; circular bins for phase):


$$\begin{array}{l} {\hat{\sigma }}_{ent}[a_{b}]=\text{exp}(\hat{H}(X_{1}^{\left(a\right)}+X_{2}^{\left(a\right)})-\hat{H}(X^{\left(a\right)})),\\ {\hat{\sigma }}_{ent}[\phi_{b}]=\text{exp}(\hat{H}(X_{1}^{\left(\phi \right)}+X_{2}^{\left(\phi \right)})-\hat{H}(X^{\left(\phi \right)})). \end{array}$$

Here, $\hat{H}(\cdot )$ is Shannon entropy from empirical histograms ( $p_{X}$ , $p_{S}$ ), as in Dembo et al. (28).

Zero-phase guarantee must be provided for guaranteeing the entropy doubling. Since $h_{b}$ is linear-phase (applied zero-phase) and $g_{\tau }$ is symmetric, the sequence $x\to x_{b}\to z_{b}\to (a_{b},\phi_{b})\to ({\tilde{a}}_{b},{\tilde{\phi }}_{b})\to (a_{b,k},\phi_{b,k})$ introduces no phase distortion (29, 30).

## Results

The mean age of the 13 female and 7 male cases was 34.7 ± 12.8 years, and the disease duration was 14.9 ± 9.6 years. The average lactate levels were calculated to be 1.8 ± 0.3 mmol/l, and the average LDH levels were calculated to be 210.1 ± 60.1 u/l.

**Figure 1:**
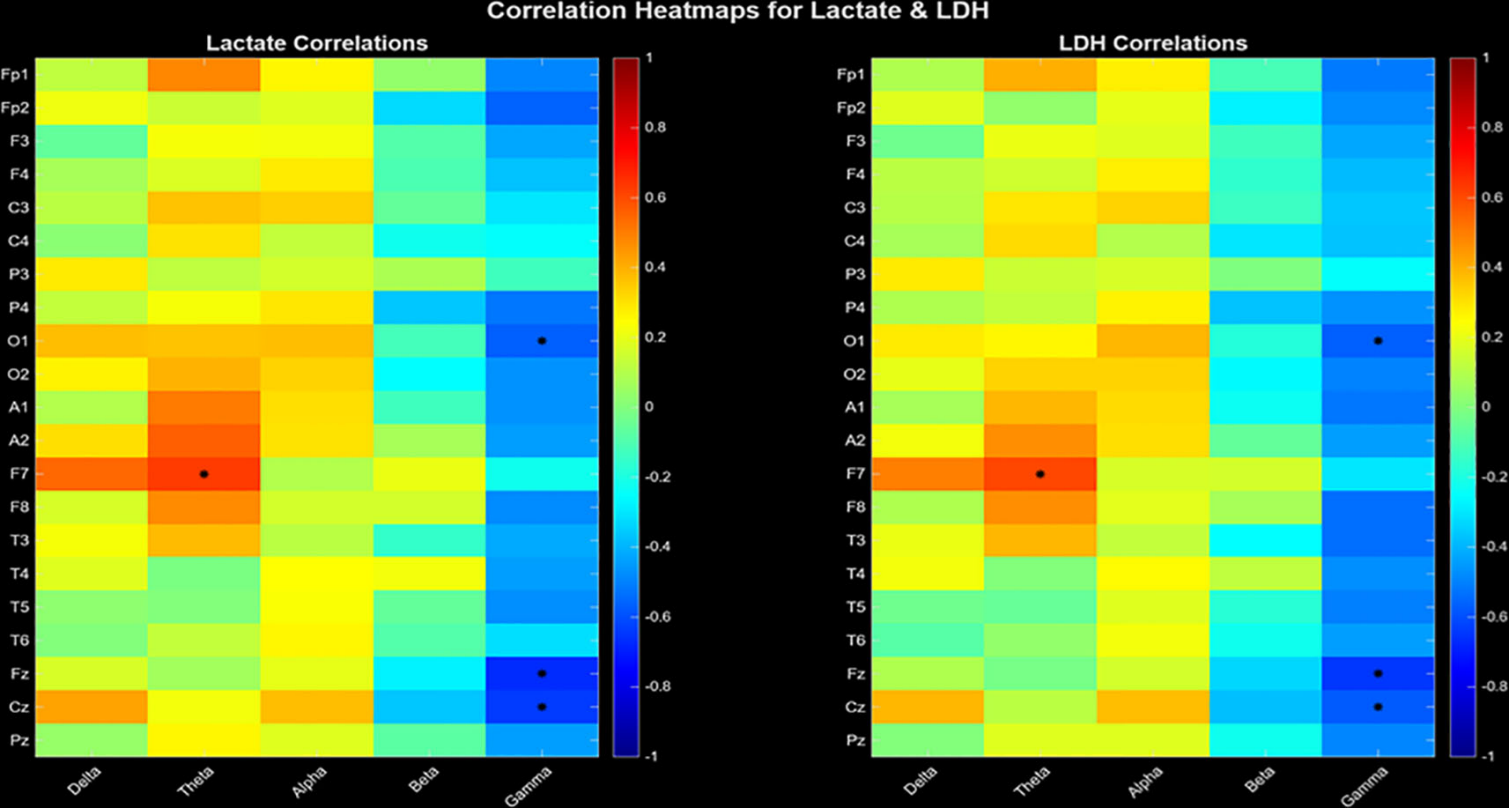
Correlation heatmaps between EEG entropy doubling (amplitude-based) and peripheral metabolic markers (Lactate, left; LDH, right). The vertical axis denotes electrode sites and the horizontal axis denotes canonical frequency bands. Warm colors indicate positive correlations, cool colors indicate negative correlations. Black asterisks mark statistically significant correlations (p< 0.05). Confidence intervals (CI = 95%) were calculated using nonparametric bootstrap resampling (N = 10,000 iterations).

Energy dysregulation includes theta and gamma frequency bands, both in relation to lactate and LDH (**Figure 1**). Each heatmap cell represents correlation coefficient (red=positive, blue=negative), with statistically significant correlations (p<0.05) marked by black asterisks. The barcode-like vertical stripes in the heatmaps correspond to frequency-specific effects: each frequency column shows a distinct pattern of correlations across electrodes. Strong red or blue ‘bars’ indicate band-driven effects, confirming that metabolic coupling is frequency-specific rather than diffuse.

Lactate and LDH levels in the F7 theta band were linearly correlated (r= 0.644, p= 0.027 ve r= 0.638, p= 0.029). A negative correlation was found between the levels of lactate (r= - 0.514, p= 0.038; r= -0.863, p< 0.001; r= -0.801, p< 0.001) and LDH levels (r= - 0.436, p= 0.042; r=.-0.684, p= 0.023; r= -0.579, p= 0.037) in the O1, Fz, and Cz gamma bands (**Table 1**). The false discovery rate (FDR) was computed using methodology described by Benjamini and Hochberg. Significant results were determined based on an FDR-adjusted *p*-value of ≤ 0.05.

**Table 1:** Correlation table.

r, p (FDR p)	Lactate	LDH
F7 Theta O1 Gamma Fz Gamma Cz Gamma	0.644, 0.027	0.638, 0.029
	-0.514, 0.038 (NS)	-0.436, 0.042 (NS)
	-0.863, 0.001	-0.684, 0.023
	-0.801, 0.001	-0.579, 0.037 (NS)

## Discussion

A major finding of this study is the strong negative correlation between serum lactate and LDH levels and gamma oscillations (**Figure 1**). Functional MRI studies have consistently shown that lactate levels increase during intense neural activation, and that EEG high-frequency power generally covarys with extracellular lactate dynamics (31). The strong correlations we observed between lactate and LDH and entropy at central electrodes may reflect changes in neuronal oxidative capacity and energy trafficking driven by lactate- and LDH-mediated metabolism. In fact, gamma oscillations are also observed as enveloped within theta oscillations, as has been demonstrated optogenetically in ex vivo experiments (32).

Gamma oscillations occur in many cortical areas during perception, psychomotor activity, and memory formation (33). They are a dialogue between glutamatergic pyramidal cells and GABAergic interneurons. Several hippocampal and neocortical GABAergic interneuron subtypes exert rhythmic perisomatic inhibition on pyramidal cells via GABA release. Rhythmic perisomatic inhibition is extensively produced by fast-spiking interneurons, such as parvalbumin-positive GABAergic basket cells. Fast-spiking interneurons possess unique electrophysiological properties, including extensive axonal arborization and high-frequency presynaptic GABA release (34). It has been suggested that the impairment of gamma oscillations during metabolic/oxidative stress originates primarily from fast-spiking interneurons rather than pyramidal cells (35). This is countered by an increase in theta activity.

In our study, there was a linear relationship between serum lactate and LDH levels and theta oscillations (**Figure 1**), which bears traces of theta-gamma coupling, suggesting a band-specific metabolic coupling. High lactate levels and associated metabolic acidosis have been associated with EEG slowing (36). This finding is consistent with meta-analyses showing that theta-beta power is associated with lactate clearance during sleep and cortical activation (37).

During low-energy network activity, lactate is an adequate substitute for glucose (38). Gamma activity, however, is attenuated at low glucose concentrations. In this rhythm, lactate can only be a supplementary fuel. Only moderate hyperexcitability, superimposed on gamma oscillations by lactate utilization, has been observed (5). This state, reflecting a type of excitation-inhibition imbalance, is suppressed by glucose utilization. The impairment in gamma oscillations occurring at low glucose concentrations is not accompanied by hyperexcitability. At this point, it is thought that the neural excitation-inhibition balance can be maintained as long as the rate of decline in glucose concentration is not too rapid. Given the dual role of lactate as a fuel and signaling molecule involved in neuroplasticity and gene regulation, EEG-metabolite relationships may reflect integrated neuroenergetic and neuroprotective processes potentially related to stress susceptibility and neurodegenerative risk.

Lactate-induced impairment of gamma and theta-gamma oscillations is associated with i) decreased neuronal excitability, ii) decreased neurotransmitter release, iii) altered postsynaptic glutamatergic and GABAergic receptor activation. This may result in i) ATP deficiency resulting from decreased glycolysis and limited lactate oxidation, ii) partial inhibition of mitochondrial respiration by neuronal NO synthesis, iii) intracellular acidification mediated by ATP hydrolysis and H-linked neuronal MCTs, iv) shifts in the cytosolic NAD/NADH ratio, v) lactate-mediated HCAR1 activation, and vi) activation of purinergic and adenosine receptors (39).

ATP synthesis by aerobic glycolysis at excitatory and inhibitory synapses, or aerobic glycolysis itself, may be essential. Lactate is less effective than glucose. Its long-term use during high-energy-cost neural network rhythms can even be potentially harmful after a certain point.

Consequently, the effects of high lactate/glucose ratios on mood and cognition appear to depend on the pathophysiological context. These exciting fundamental concepts and their clinical implications require comprehensive collaborative studies involving morphological, biochemical, electrophysiological, and neuroimaging methods.

Our limitation is that correlation cannot confirm causation: i) EEG captures millisecond-scale activity, while lactate- and LDH-related changes are slower, which future simultaneous fMRI-EEG protocols will clarify temporal coupling. ii) EEG energy measurements likely combine multiple processes. Complementary studies using MCT inhibitors or LDH isoform assays may be able to distinguish between the contributions of lactate transport and its conversion.

In this study, we asked a simple, yet valid and important question. This is true because we found highly significant correlations. This is important because it was investigated for the first time in patients diagnosed with bipolar disorder. We demonstrated a relationship between peripheral metabolic markers (lactate, LDH) and EEG signal complexity. The lack of a control group should have been emphasized as a significant limitation. At this point, we would like to point out that our patients being in remission would bring our findings closer to those of healthy controls.

Our findings are also intriguing in terms of the connections revealed along the brain-body axis. The fact that our patients were in remission brings the associations we found closer to those found in the healthy population. Future studies are intriguing, given the nature and severity of depressive and manic episodes. Both peripheral metabolic measurements and EEG changes, and particularly the relationship between them, are potential biomarkers reflecting mitochondrial dysfunction in bipolar disorder and illness episodes.

## Data Availability

The raw data supporting the conclusions of this article will be made available by the authors, without undue reservation.
